# Evidence of Experimental Vertical Transmission of Emerging Novel ECSA Genotype of Chikungunya Virus in *Aedes aegypti*

**DOI:** 10.1371/journal.pntd.0002990

**Published:** 2014-07-31

**Authors:** Ankita Agarwal, Paban Kumar Dash, Anil Kumar Singh, Shashi Sharma, Natarajan Gopalan, Putcha Venkata Lakshmana Rao, Man Mohan Parida, Paul Reiter

**Affiliations:** 1 Division of Virology, Defence R and D Establishment, Gwalior, Madhya Pradesh, India; 2 Division of Vector Management, Defence R and D Establishment, Gwalior, Madhya Pradesh, India; 3 Insects and Infectious Disease Unit, Institut Pasteur, Paris, France; United States Army Medical Research Institute of Infectious Diseases, United States of America

## Abstract

**Background:**

Chikungunya virus (CHIKV) has emerged as one of the most important arboviruses of public health significance in the past decade. The virus is mainly maintained through human-mosquito-human cycle. Other routes of transmission and the mechanism of maintenance of the virus in nature are not clearly known. Vertical transmission may be a mechanism of sustaining the virus during inter-epidemic periods. Laboratory experiments were conducted to determine whether *Aedes aegypti*, a principal vector, is capable of vertically transmitting CHIKV or not.

**Methodology/Principal Findings:**

Female *Ae. aegypti* were orally infected with a novel ECSA genotype of CHIKV in the 2^nd^ gonotrophic cycle. On day 10 post infection, a non-infectious blood meal was provided to obtain another cycle of eggs. Larvae and adults developed from the eggs obtained following both infectious and non-infectious blood meal were tested for the presence of CHIKV specific RNA through real time RT-PCR. The results revealed that the larvae and adults developed from eggs derived from the infectious blood meal (2^nd^ gonotrophic cycle) were negative for CHIKV RNA. However, the larvae and adults developed after subsequent non-infectious blood meal (3^rd^ gonotrophic cycle) were positive with minimum filial infection rates of 28.2 (1∶35.5) and 20.2 (1∶49.5) respectively.

**Conclusion/Significance:**

This study is the first to confirm experimental vertical transmission of emerging novel ECSA genotype of CHIKV in *Ae. aegypti* from India, indicating the possibilities of occurrence of this phenomenon in nature. This evidence may have important consequence for survival of CHIKV during adverse climatic conditions and inter-epidemic periods.

## Author Summary

Although vertical transmission of arboviruses has been recognized for nearly a century, rates of transmission in laboratory experiments are low and their significance in terms of survival of virus during periods of low transmission appears debatable. Recently, major urban outbreaks of chikungunya have been recorded in many parts of Asia, Africa, and Europe. The occurrence of random sporadic cases of the disease in years following a major outbreak prompted us to investigate whether these might be attributable to survival of the virus by vertical transmission. Our experiments were designed to test two hypotheses: (1) The development of an egg-batch derived from an infectious blood meal is too rapid for the infection to reach ovaries; (2) The enormous distension of the membrane enveloping ovaries and ovarioles following oviposition, might facilitate virus penetration. We conclude that after the infected blood meal, oogenesis and oviposition were complete before virus had disseminated to infect the ovaries. Because similar experiments with infection in first gonotrophic cycle did not lead to infected progenies, it is presumed that expanded parous ovaries might support efficient infection. Therefore, it may be concluded that vertical transmission is a more common phenomena in mosquitoes during subsequent gonotrophic cycles following arboviral infection.

## Introduction

Chikungunya virus (CHIKV) (genus *Alphavirus*, family *Togaviridae*) is a mosquito-borne pathogen, native to Africa that is transmitted between non human primates, mainly by forest dwelling *Aedes* species. The virus is also widespread as an urban infection throughout the old world tropics and subtropics, transmitted by two species of mosquito- *Aedes aegypti* and *Ae. albopictus*, both closely associated with the human peridomestic environment [1]. In Asia, the *Ae. aegypti* mosquitoes are primarily responsible for the maintenance of urban cycle, while in Africa, CHIKV transmission involves a sylvatic cycle, primarily with *Ae. furcifer* and *Ae. africanus* mosquitoes [2]. Autochthonous cases have also occurred in Europe, most notably in 2007 in an epidemic in northeast Italy that affected nearly 300 people [3]. In this case the vector was *Ae. albopictus*, an invasive species that is rapidly expanding its distribution in Europe and is already present in at least 27 countries.

In humans, chikungunya fever is a self-limiting illness. In acute phase, it may involve some or all of the following: sudden onset of fever, headache, fatigue, nausea, vomiting, rash, myalgia and severe polyarthralgia. Symptoms may last up to 10 days, but crippling arthralgia can persist for months, even years in some patients [4]. CHIKV genome consists of 5′ capped positive sense single-strand RNA of ∼11.8 kb that harbors a poly (A) tail in its 3′ end. The genome is composed of two open reading frames (ORFs) embedded between non-translated regions (5′ NTR and 3′ NTR). The ORF located at the 5′ end of the genome encodes a polyprotein precursor of nonstructural proteins (nsP1, nsP2, nsP3, nsP4) with replicative and proteolytic activities. The second ORF encodes the polyprotein precursor of the structural proteins (C, E1, E2) [5].

Vertical transmission is the passage of virus between generations via the egg stage. Virus that infects the ovaries must persist through the larval instars, survive histolysis in the pupal instar and continue through to the adult stage [6]. Vertical transmission is considered to be a primary means by which some arboviruses are maintained during adverse environmental conditions. During this period, the arthropod hosts are either inactive or unable to survive, thus acting as a mechanism for virus persistence in environments where amplifying hosts are temporarily absent or immune. Because the *Aedes* eggs are desiccation resistant, these can survive for longer durations, leading to the possibility of persistence of CHIKV in the eggs [7]. The mechanisms responsible for prevalence of CHIKV during unfavourable periods, especially during winter seasons are unknown. So, vertical transmission is considered as an unresolved issue that has important bearing on the persistence of virus in periods when horizontal transmission is low or non-existent.

Low rates of vertical transmission of the three main groups of mosquito-borne arboviruses- flaviviruses, alphaviruses, and bunyaviruses have been demonstrated in the field [8]–[10]. The existence of vertical transmission has also been demonstrated experimentally in these three groups. [11]–[14]. Among the alphaviruses, Ross River virus, Sindbis virus, western equine encephalomyelitis virus, and CHIKV have been isolated from adult *Aedes* species reared from larvae collected from natural habitats, confirming existence of natural vertical transmission [15]–[20]. Lindsay and coworkers isolated Ross River virus from wild-caught male *Aedes* mosquitoes, further providing evidence of natural vertical transmission [21]. However, to the best of our knowledge, there is no evidence of experimental vertical transmission among alphaviruses other than some conflicting reports in CHIKV [13], [22], [23].

In reviewing the literature on laboratory infections we noted that in nearly all studies, the infective blood meal was given to nulliparous mosquitoes [11], [12] and detection of virus was limited to the offspring of the first gonotrophic cycle, whereas, when studies continued through subsequent cycles, rates of vertical transmission were found much higher [11], [24], [25]. We speculated that the difference could be attributable to two factors:(i) the first batch of eggs is laid several days before virus has begun rapid replication after passing via the gut wall into the hemolymph, and/or (ii) the enormous increase in the volume of the ovaries during oogenesis might increase its permeability to virus. We explored these possibilities by investigating the occurrence of vertical transmission in experimentally infected *Ae. aegypti*, the principal vector of CHIKV.

## Materials and Methods

### Viruses and mosquitoes

CHIKV belonging to novel Indian Ocean lineage (IOL) of ECSA genotype, obtained from an epidemic in India in 2006 (DRDE06) (GenBank Accession No. EF210157). Initially it was isolated in BHK-21 cells and subsequently passaged in C6/36 cells. The virus is maintained at Virology Division, DRDE, Gwalior. In the present study, it was used at passage level 10 in C6/36 cells. Titre was found to be 10^8^ PFU/ml through plaque assay [26] in Vero cells (African green monkey kidney cells). The virus was aliquoted and stored in −80°C until use.

*Ae. aegypti* used in this study, were collected from Gwalior district, Madhya Pradesh, India in 2005 and maintained as laboratory colony in Vector Management Division, Defence Research and Development Establishment (DRDE), Gwalior at 27±2°C with 70% relative humidity and 14∶10 light∶dark photo period. Adult mosquitoes were provided with 10% sucrose solution soaked in cotton pads and larvae were provided with 2–3 yeast tablets per day in a pan containing tap water.

### Oral infection of mosquitoes

**Figure 1:**
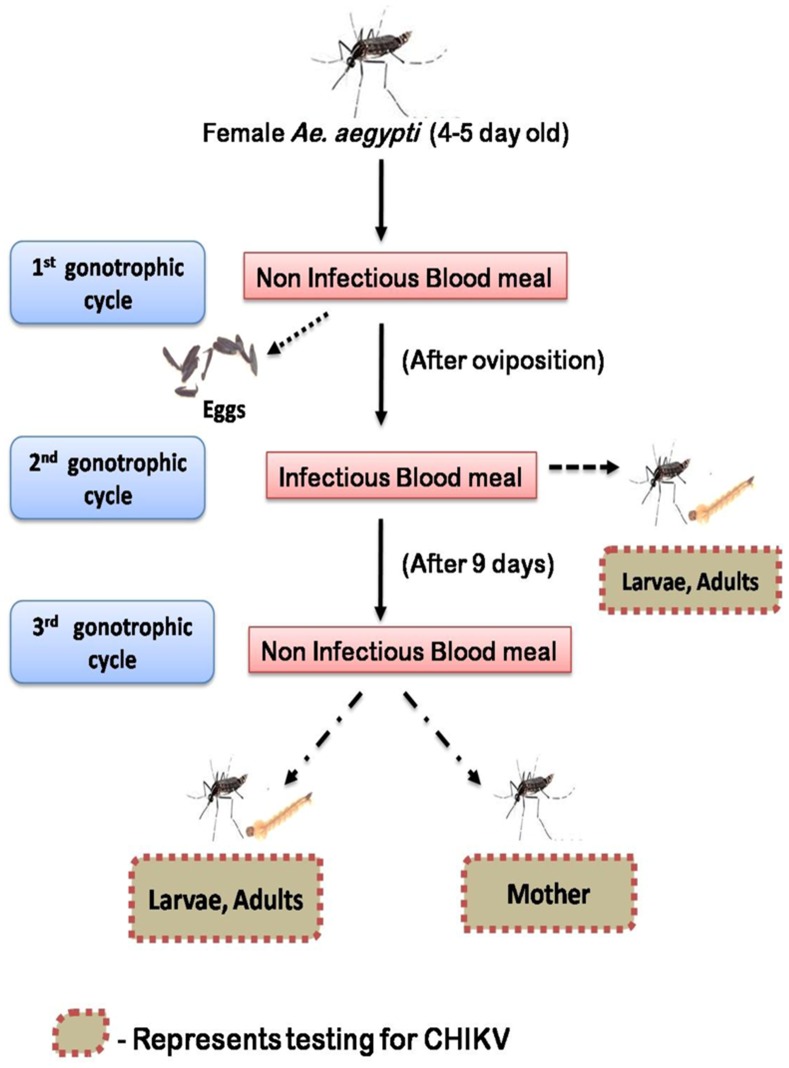
Schematic representation of the experimental design.

The female *Ae. aegypti* mosquitoes were provided with three serial blood meals during this experiment to investigate vertical transmission. Schematic representation of the experimental design is shown in Fig. 1.

#### I Non infectious blood meal

First, a non infectious blood meal (only rabbit blood) was given to 4–5 days old female *Ae. aegypti*. Fully engorged females were separated and allowed to lay eggs. The eggs thus obtained were not utilized for this experiment.

#### II Infectious blood meal

Following oviposition from the 1^st^ non infectious blood meal, these *Ae. aegypti* were starved for 24 hours before providing infectious blood meal in a BSL-3 laboratory. Around 50 of these mosquitoes kept in plastic boxes with mesh covers, were allowed to feed for 45 minutes through a hemotek feeding membrane (Discovery workshops, Accrinton, UK) covering the base of a glass feeder containing the blood-virus mixture. The temperature of the blood meal was maintained at 37°C through a water circulator. The infectious blood meal was prepared by adding 1 ml of viral suspension (10^8^ PFU/ml) in 2 ml of washed rabbit erythrocytes to a final concentration of 3.3×10^7^ PFU/ml. ATP was added to a final concentration of 5 mM as a phagostimulant. After 45 minutes of feeding, mosquitoes were cold anaesthetized and sorted on ice. 20 fully engorged females were transferred to each small cardboard container and maintained with 10% sucrose for 9 days. The eggs (obtained within 3–4 days) following this infectious blood meal were further processed.

#### III Non infectious blood meal

On day 10 post infection, the mosquitoes were provided with a non-infectious blood meal (only rabbit blood). Fully engorged females were separated and kept individually in conical tubes (50 ml Falcon tubes) containing moistened filter paper and cotton at the bottom. The eggs laid were then further processed.

### Testing of parental females for disseminated infection

At day 14 post infection, 10 *Ae. aegypti* parental females (after laying eggs from 3^rd^ gonotrophic cycle) were randomly selected and their midgut, legs & wings were tested for the presence of CHIKV to analyze infection and dissemination status.

### Processing of larvae and adults

The eggs obtained after infectious blood meal and second non-infectious blood meal were reared under standard laboratory conditions i.e. 27±2°C at 70% relative humidity. The 4^th^ instar larvae and 4–5 days old adults were screened for the presence of virus. The larvae and adults were processed in pools (≤20/pool).

### Extraction of RNA

Each pool of adults and larvae were homogenized in 2 ml tubes with 800 µl of Eagles Minimum Essential Medium (EMEM) (Sigma, St. Louis, USA) and stainless steel beads in Tissuelyser LT (Qiagen, Hilden, Germany). The homogenate was clarified by centrifugation at 4500× *g* for 10 minutes. 140 µl of supernatant was used to extract RNA using QIAamp viral RNA mini kit (Qiagen, Hilden, Germany) according to the manufacturer's protocol. The RNA was finally eluted in 50 µL elution buffer and stored at −80°C until use.

### CHIKV specific SYBR Green-I based Real time RT-PCR

CHIKV specific SYBR Green I based one step real time quantitative RT-PCR targeting to E1 gene was performed to screen the presence of CHIKV RNA in test samples [27]. Briefly, the quantitative RT-PCR was carried out using SS III Platinum one step qRT-PCR kit (Invitrogen, Carlsbad, USA) in Mx3005P system (Stratagene, La Jolla, USA). Samples were assayed in a 25 µL reaction volume containing 12.5 µL of 2× master mix, 0.125 µL (0.25 µmol) each of forward and reverse primer, 0.25 µL of enzyme mix comprising of Taq DNA polymerase and MMLV Reverse transcriptase, 9.5 µL of nuclease free water and 2.5 µL of RNA. The thermal profile comprised of 30 min of reverse transcription at 50°C, 10 min of polymerase activation at 95°C, followed by 40 cycles of PCR at 95°C for 30 s, 55°C for 60 s, and 72°C for 30 s. Following amplification, a melting curve analysis was performed with the melting curve analysis software of the Mx3005P according to the instructions of manufacturer. Positive and negative template control was also included along side in all experiments.

### Statistical analysis

CHIKV RNA mean titre in larvae and adults were analyzed. Comparison between two groups was performed by 2 tailed unpaired t-test using GraphPad Prism software (Version 6.04).

## Results

### Detection of CHIKV in parental females

CHIKV RNA was detected in body, legs & wings of all the 10 randomly selected female *Ae. aegypti* that were provided with an infectious blood meal. This indicates 100% infection and dissemination of virus at day 14 post infection. The mean titre of CHIKV RNA in midgut, legs & wings of female *Ae. aegypti* was found to be 10^6.4^±10^1.4^ and 10^6.0^±10^1.0^ per reaction respectively.

### Vertical transmission rates in larvae and adults

The total number of larvae and adults obtained after infectious blood meal were 230 and 485 respectively. All these larvae and adults were found to be negative for CHIKV. The total number of larvae and adults obtained after second non infectious blood meal were 284 and 693 respectively. These were divided and processed in pools (≤20/pool). Out of a total 30 pools of larvae, 8 pools were found positive. Out of 33 pools of adults, 14 were found positive.

Minimum infection Rate (MIR) was calculated by the following formula: No. of positive pools/No. of individuals tested X 1000 [25]. The minimum infection rates achieved for larvae and adults were 28.2 and 20.2 respectively. MIR is also expressed as ratio i.e. Proportion of positive mosquitoes by uninfected mosquitoes. Thus the ratio was found to be 1∶35.5 for larvae and 1∶49.5 for adults [25] (Table 1).

**Table 1:** Rate of vertical transmission of Chikungunya virus in 3^rd^ gonotrophic cycle.

*Stage Examined*	*Number Analyzed*	*Positive pools/tested pools*	*MIR*	*Ratio*
Larvae	284	8/30	28.2	1∶35.5
Adults	693	14/33	20.2	1∶49.5
Total	977	22/63	22.5	1∶44.4

Ratio = Proportion of positive mosquitoes by uninfected mosquitoes.

MIR (Minimum infection rate) expressed as Ratio.

The results indicated above are cumulative of three experiments. Because the feeding efficiency was low and fewer mosquitoes underwent oviposition with successive gonotrophic cycles, fewer larvae and adults were obtained.

### Quantification of CHIKV in larvae and adults

**Figure 2:**
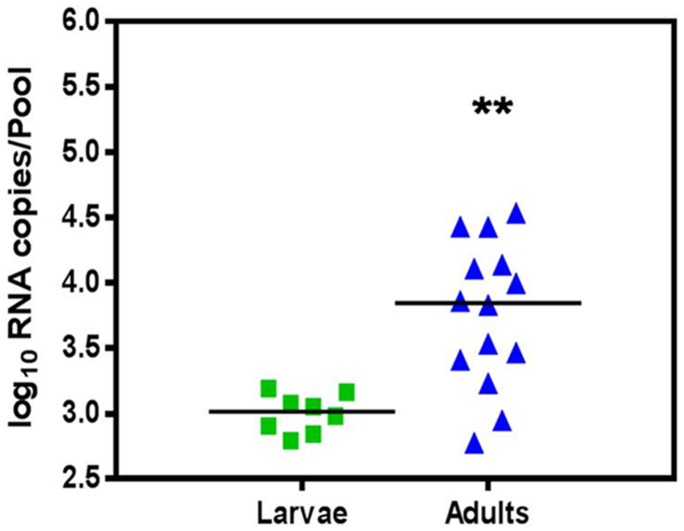
Vertical transmission of Chikungunya virus in *Ae. aegypti* as determined by measuring log_10_ RNA copies/pool of larvae and adults by real time RT-PCR. Horizontal line represent mean value for each group. Asterisks are indicating significant differences between the two groups (**p<0.01).

The CHIKV RNA titre as determined by real time RT-PCR in larvae was 10^2.8^ to 10^3.2^ (10^3.0^±10^0.1^). The CHIKV RNA titre in adults was 10^2.8^ to 10^4.5^ (10^3.8^±10^0.6^) (Fig. 2). Significant difference was observed between the two groups (p = 0.0012) with t = 3.788 and df = 20. The result indicated the amplification of virus following transition from larvae to adult stage.

## Discussion

Since the emergence of the novel ECSA genotype of CHIKV in 2005, widespread outbreaks have been reported in many parts of Africa, Asia and Europe. Intense speculation has been generated regarding different transmission pattern of this emerging virus. So far horizontal transmission is the only method clearly linked to transmission of CHIKV. However, natural vertical transmission of CHIKV has been recently reported from India [17], Reunion Island [18], Thailand [19] and Madagascar [20]. This mode of transmission was recorded in both *Ae. albopictus* and *Ae. aegypti* mosquitoes. In contrast, the vertical transmission experiments in laboratory have generated conflicting reports [13], [22], [23]. Though a very high rate of vertical transmission (18–62%) was recorded among different species of *Aedes* mosquito in one experimental study [13], two other studies failed to document existence of vertical transmission [22], [23]. A number of variable factors including the viral assay methods, mosquito species, viral isolate, day of sampling and blood meal feeding might have contributed to these conflicting results. Thus, the present study was carried out to investigate the existence of vertical transmission of novel IOL lineage of ECSA genotype of CHIKV in *Ae. aegypti* from India, because it is widely reported to be the major *Aedes* species in northern India [28].

Our earlier experiment using the same CHIKV isolate and *Ae. aegypti*, revealed that the viral dissemination started from day 3 post infection onwards and reached its peak on day 10 post infection [27]. Other replication kinetics experiments of CHIKV in *Ae. aegypti* clearly revealed high viral dissemination from day 8–12 post infection [29]. These observations guided us to provide a non infectious blood meal at day 10 post infection to maximize infected eggs. The infectivity status of the parental female *Ae. aegypti* on day 14 post infection was confirmed through the detection of CHIKV RNA in the midgut, indicating successful CHIKV replication following oral infection. Further, the viral RNA titers in legs and wings of parental females also suggested efficient dissemination of virus within the mosquito. This was considered as an evidence of dissemination of CHIKV to various secondary organs, presumably ovaries also. In our experiment, larvae and adults developed directly from the eggs after infectious blood meal were found negative for CHIKV RNA. This was primarily due to shorter gonotrophic cycle, where the eggs were laid within 2–3 days of ingestion of infectious blood meal, prior to the adequate dissemination of the virus to ovaries and oviduct. However, larvae and adults developed after the subsequent non infectious blood meal (3^rd^ gonotrophic cycle) were found positive for CHIKV RNA, suggesting an adequate incubation period is required for viral dissemination to the ovaries. Considerable amount of viral particles was earlier demonstrated in the ovaries on 6^th^ day post infection, indicating possibility of vertical transmission of CHIKV [30]. The result obtained by Anderson et al. also suggested the importance of extrinsic incubation period on vertical transmission. In their experiment, F1 progeny developed from eggs laid after 1^st^ infectious blood meal were negative for West Nile virus, whereas progeny obtained after subsequent blood meals were positive [31]. Similar observations were also reported for LaCrosse virus [32], Kunjin virus [33] and yellow fever virus [34].

Comparison with recent laboratory vertical transmission results, where progenies were tested during 1^st^ and 2^nd^ gonotrophic cycle following infectious blood meal of ECSA genotype of CHIKV revealed negative [23] to very low positive results (0.43%) [35]. Similar results were also reported with Asian genotype CHIKV and Indian *Aedes* mosquitoes in consecutive gonotrophic cycles [22]. Due to these negative results, we tried to evaluate the effect of infection in 2^nd^ gonotrophic cycle and continued the experiments till 3^rd^ gonotrophic cycle, which resulted in high positivity. The infection of expanded parous ovaries with massive stretching of peritrophic membrane might have facilitated better viral infection of the oocytes. In contrast, Vazeille and co-authors [23] offered a non infectious blood meal prior to an infected one, however, the evaluation of progenies were only restricted to 2^nd^ gonotrophic cycle, that failed to document evidence of vertical transmission. The follow up study to the next gonotrophic cycle, which was not performed, might have contributed to the negative result in their study.

The minimum infection rates achieved for larvae and adults were 28.2 (1∶35.5) and 20.2 (1∶49.5) respectively. Such high MIR in vertical transmission experiments was also described earlier in Japanese encephalitis and West Nile viruses [11], [12], [36]. High MIR for West Nile virus (up to 31.1) was observed among male *Culex* mosquitoes collected in Connecticut, New Haven, USA [9]. These high MIR rates are likely to be indicator of potential outbreaks.

The demonstration of high viral titer of CHIKV in midgut, legs and wings of Indian *Ae. aegypti*, implicate this species as a major vector of CHIKV (IOL of ECSA genotype). Further, vertical transmission is a plausible survival mechanism for the virus, enabling the virus to be endemic during inter-epidemic periods. In view of the increasing reports regarding identification of natural vertical transmission of CHIKV from different continents, the conflicting results from artificial vertical transmission experiments may be laboratory artifacts.

### Conclusion

The identification of vertical transmission of CHIKV in both natural and experimental settings, confirms the existence of this transmission pattern. The present study indicated that vertical transmission is a more common phenomena in mosquitoes during subsequent gonotrophic cycles following an arboviral infection. In view of the desiccation resistance nature of *Aedes* eggs, vertical transmission is likely to facilitate the persistent survival of virus during unfavourable inter-epidemic periods. This survival of virus has immense epidemiological implication further enhancing the risk of potential future outbreaks.
